# Supplementary material for: Venomix: a simple bioinformatic pipeline for identifying and characterizing toxin gene candidates from transcriptomic data
Source: PeerJ. 2018 Jul 31;6:e5361. doi: 10.7717/peerj.5361 (PMC6074769; doi:10.7717/peerj.5361)
Supplement: Supplemental Information 3 [file peerj-06-5361-s003.gz › FinalOutput_E-6/Acidic_phospholipase_A2_daboiatoxin_B_chain_6/finaltree.pdf]

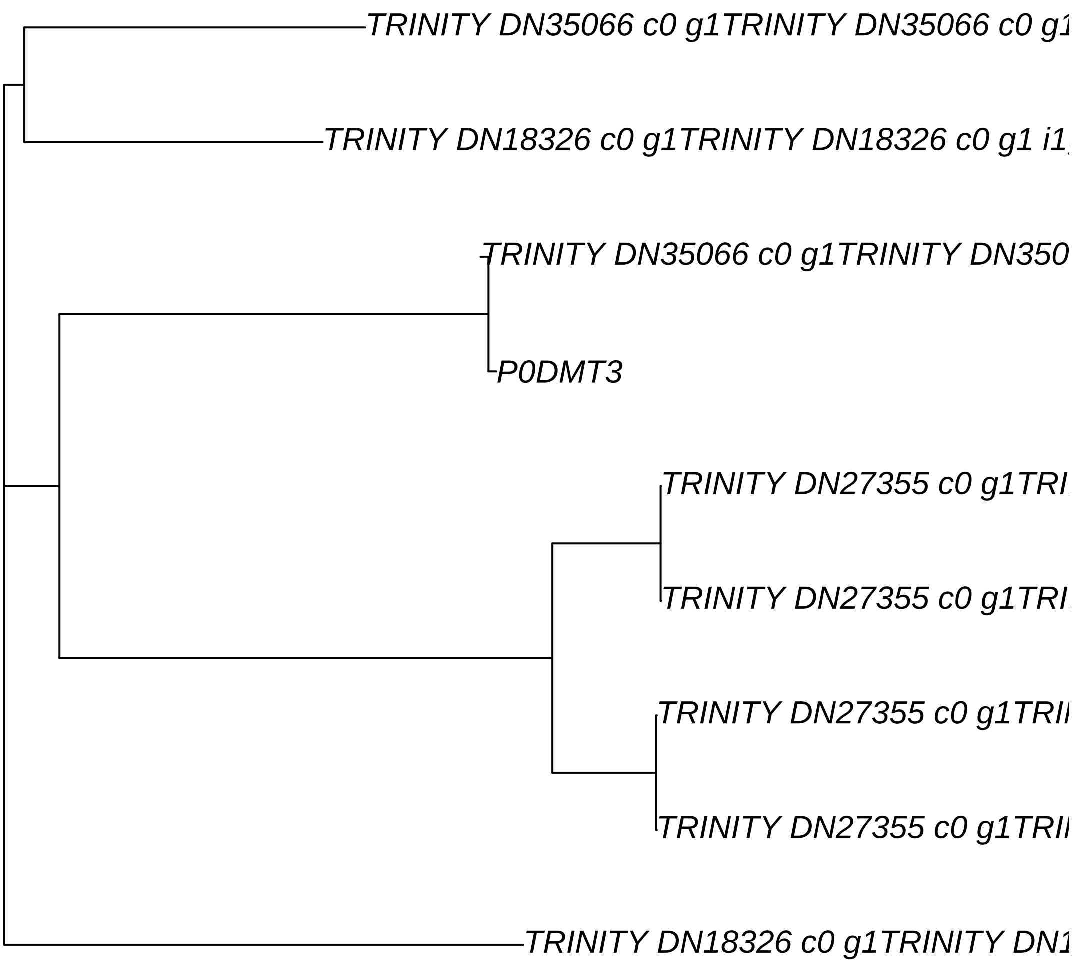
TRINITY DN35066 c0 g1TRINITY DN35066 c0 g
TRINITY DN18326 c0 g1TRINITY DN18326 c0 g1 i1
TRINITY DN35066 c0 g1TRINITY DN350
P0DMT3
TRINITY DN27355 c0 g1TRI
TRINITY DN27355 c0 g1TRI
TRINITY DN27355 c0 g1TRI
TRINITY DN27355 c0 g1TRI
TRINITY DN18326 c0 g1TRINITY DN1
